# The classic signalling and trans‐signalling of interleukin‐6 are both injurious in podocyte under high glucose exposure

**DOI:** 10.1111/jcmm.13314

**Published:** 2017-09-07

**Authors:** Chun‐Tao Lei, Hua Su, Chen Ye, Hui Tang, Pan Gao, Cheng Wan, Fang‐Fang He, Yu‐Mei Wang, Chun Zhang

**Affiliations:** ^1^ Department of Nephrology Union Hospital Tongji Medical College Huazhong University of Science and Technology Wuhan China

**Keywords:** interleukin‐6, diabetic kidney disease, podocyte, STAT3

## Abstract

Interleukin‐6 (IL‐6) is a multifunctional cytokine that employs IL‐6 classic and trans‐signalling pathways, and these two signal channels execute different or even opposite effects in certain diseases. As a cardinal event of diabetic kidney disease (DKD), whether the podocyte abnormalities are associated with IL‐6 signalling, especially classic or trans‐signalling respectively, remains unclear. In this study, we identified that the circulatory IL‐6, soluble IL‐6R (sIL‐6R) and soluble glycoprotein 130 (sgp130) levels are elevated in patients with DKD. The expressions of membrane‐bound IL‐6R (mIL‐6R), sIL‐6R and gp130 are enhanced in kidney cortex of diabetic mice accompanying with activated STAT3 by tyrosine 705 residue phosphorylation, while not serine 727. Above data infer both classic signalling and trans‐signalling of IL‐6 are activated during DKD. In cultured podocyte, high glucose (HG) up‐regulates the expression of mIL‐6R and gp130, as well as STAT3 tyrosine 705 phosphorylation, in a time‐dependent manner. Entirely blocking IL‐6 signalling by gp130 shRNA, gp130 or IL‐6 neutralizing antibodies attenuates HG‐induced podocyte injury. Interestingly, either inhibiting IL‐6 classic signalling by mIL‐6R shRNA or suppressing its trans‐signalling using sgp130 protein dramatically alleviates HG‐induced podocyte injury, suggesting both IL‐6 classic signalling and trans‐signalling play a detrimental role in HG‐induced podocyte injury. Additionally, activation of IL‐6 classic or trans‐signalling aggravates podocyte damage in vitro. In summary, our observations demonstrate that the activation of either IL‐6 classic or trans‐signalling advances podocyte harming under hyperglycaemia. Thus, suppressing IL‐6 classic and trans‐signalling simultaneously may be more beneficial in podocyte protection and presents a novel therapeutic target for DKD.

## Introduction

Diabetic kidney disease (DKD) is a critical microvascular complication of diabetic mellitus (DM) and one of the primary causes for end stage renal disease (ESRD). With the impressive increased incidence of DM, there has been a strikingly rise of DKD and ESRD in the worldwide 1, 2, 3. Although multiple approaches targeting on controlling glucose or inhibition of renin–angiotensin system (RAS) have been applied in clinical patients, there is no satisfactory strategy available to efficiently prevent the onset and progression of DKD presently. Podocyte injury is one of the major pathological events that involved in the early and central stage of DKD 4, 5. However, the mechanism implicated in podocyte injury has not been fully clarified yet, and comprehensive understanding of the mechanism of podocyte injury is critical for DKD efficacious intervention.

Interleukin‐6 (IL‐6) is a well‐described pleiotropic cytokine belonging to the glycoprotein 130 (gp130) family of cytokines, and it not only promotes T‐cell activation, B‐cell differentiation and the cell population expansion during inflammatory response 6, but also plays an important role in insulin resistance, lipid metabolism, mitochondrial activities and other situations 7. Growing evidences indicate that elevated serum inflammatory cytokines positively correlate with the onset and progression of DM microvascular complication including DKD 8, 9, 10, 11, 12. Conspicuously, IL‐6 abundance positively parallels with the extent of proteinuria 13 and the reduction of glomerular filtration rate (GFR) in type 2 diabetic patients 14. High glucose stimulates IL‐6 generation from various renal resident cells which contributes to cellular and tissue injury 15, 16, 17, 18, 19. IL‐6 has two different activation pathways to initiate intracellular cascades and events *via* a specific membrane‐bound receptor (mIL‐6R) and a soluble form of IL‐6R (sIL‐6R), which are termed as classic and trans‐signalling of IL‐6, respectively. IL‐6 classic and trans‐signalling are considered to mediate different biological processes under certain circumstances. Notably, podocyte is the only glomerular resident cell that expresses mIL‐6R and can response to both classic and trans‐signalling of IL‐6 20, 21, 22. However, the respective role of IL‐6 classic and trans‐signalling in HG‐induced podocyte injury has not been clearly elucidated yet.

It is well established that Janus‐activated kinase (JAK) / signal transducers and activator of transcription 3 (STAT3) is the most important signalling cascade involving in IL‐6 transduction and that is up‐regulated in glomeruli and tubular area of DKD 23. It is widely accepted that phosphorylation of tyrosine residue (Tyr 705) is critical for the transactivation function of STAT3; however, the function of serine phosphorylation form (Ser 727) of STAT3 is arguable 24. It has been shown that these two different phosphorylation forms of STAT3 may mediate distinct biological functions 25, 26. As we know, IL‐6 classic signalling and trans‐signalling activate intracellular signalling *via* gp130 cascade but exhibit different properties in diseases including renal disorders 6, 27, 28, 29; therefore, we speculate whether the different phosphorylation forms of STAT3 are responsible for the distinct pathophysiological events of IL‐6 classic and trans‐signalling.

In this study, we investigated whether and how IL‐6 classic and trans‐signalling involved in HG‐induced podocyte injury. Our observations demonstrate that both IL‐6 classic signalling and trans‐signalling accelerate podocyte and glomeruli damage during hyperglycaemia. Completely inhibition of IL‐6 cascade or separately blocking its classic or trans‐signalling all can alleviate HG‐induced podocyte injury *via* disrupting the phosphorylation of STAT3 on Tyr 705, and irrelevant to the Ser 727 phosphorylation form.

## Materials and methods

### Ethics statement

All human samplings and animal experimental procedures performed in this study were approved by the Ethics Committee of Huazhong University of Science and Technology. The patients diagnosed with DKD were enrolled, and blood samples were obtained from Department of Nephrology and Endocrinology of Wuhan Union Hospital. The control samples were collected from the physical examination centre, matched with gender and age. Mice were treated humanely, and all the procedures were carried out in conformity with the guidelines for use and care of laboratory animals of National Institutes of Health (NIH), and ratified by the Animal Care and Use Committee (ACUC) of Tongji Medical College.

### Enzyme‐linked immunosorbent assay (ELISA) analysis

Peripheral venous blood was collected after an overnight fasting. The serum samples were aliquoted and stored in −80 ^◦^C freezers until analysed. IL‐6, sIL‐6R and sgp130 levels were measured using human IL‐6 (Elabscience, Wuhan, China) and sIL‐6R and sgp130 (SenBeiJia Biotechnology, Nanjing, China) ELISA kits according to the manufacturer's instructions.

### Animals

Eight‐week‐old C57BL/6 mice were treated with a single intraperitoneal injection of streptozotocin (STZ, 150 mg/kg, BOSTER, Wuhan, China) in citrate buffer to establish diabetic model. Control C57BL/6 mice were treated with only citrate buffer. Blood glucose was monitored weekly by glucometer readings. Only the mice with stable serum glucose levels higher than 16.7 mmol/l were included in the following experiments 5. 12 weeks later, the mice were killed and kidneys were collected.

### Cell culture and treatment

An immortalized human podocyte cell line was cultured and maintained as described previously 5. Briefly, cells were routinely cultured in RPMI1640 medium supplemented with 10% foetal bovine serum (FBS), 100 U/ml penicillin and 100 U/ml streptomycin. Firstly, cells were incubated at 33°C for proliferation, and after reached at 70% confluence, the cells were transferred to 37°C for 2 weeks to allow differentiation. Differentiated podocytes were exposed to media containing high glucose (HG, final glucose concentration 30 mmol/l) or 19.9 mmol/l mannitol as osmotic control.

After individual pre‐treatment with gp130 antibody (2 μg/ml, R&D Systems, Minneapolis, MN, USA), IL‐6 antibody (1 μg/ml, R&D Systems, Minneapolis, MN, USA), recombinant sgp130 (1 μg/ml, R&D Systems, Minneapolis, MN, USA), recombinant human IL‐6 (20 ng/ml, Peprotech, Rocky Hill, NJ, USA) or complex of IL‐6 and recombinant human soluble IL‐6R protein (30 ng/ml, R&D Systems, Minneapolis, MN, USA), podocytes were exposed to HG or osmotic control for 24 hrs.

### shRNA transfection

For RNA interference, recombinant lentivirus vector harbouring a short‐hairpin RNA sequence targeting on IL‐6R (IL‐6R shRNA) and gp130 (gp130 shRNA) was obtained from JikaiGene (Shanghai, China) and the scrambled shRNA was used as control. Podocytes were transiently transfected with target shRNA or scrambled shRNA according to the manufacturer's instruction. 48 hrs after transfection, the podocytes were exposed to HG or osmotic control for 24 hrs, and then, cells were harvested for further experiments.

### Western blot analysis

Proteins were extracted from mouse kidney cortex and cultured cells with RIPA lysis buffer (Beyotime, Jiangsu, China). Protein concentration was measured by BCA Protein Assay kit (Bio‐Rad, Hercules, CA, USA). After boiling for 5 min. at 95°C in 5× loading buffer, an equal amount of protein (40 μg) was separated *via* 8% SDS‐PAGE and electrotransferred to polyvinylidene difluoride membranes (Merck Millipore, Darmstadt, Germany). The membranes were routinely processed *via* blocking with 5% milk or 5% BSA and were incubated overnight with primary antibodies, followed by a 1‐hr incubation with horseradish peroxidase‐conjugated secondary antibodies. Immunoreactive proteins were detected using an Enhanced Chemiluminescence (ECL) Kit (Amersham). The antibodies used in this study were as follows: anti‐mIL‐6R (rabbit, 1:200), anti‐gp130 (rabbit, 1:1000), anti‐phospho‐STAT3 Tyr705 (mouse, 1:200) and β‐actin (mouse, 1:10,000) from Santa Cruz Biotechnology (Santa Cruz, CA, USA); anti‐sIL‐6R (mouse, 1:500; Abcam, Cambridge, MA, UK); and anti‐desmin (rabbit, 1:1000), anti‐phospho‐STAT3 Ser727 (rabbit, 1:1000) and anti‐STAT3 (rabbit, 1:1000) from Bioworld Technology (Louis Park, MN, USA). Densities of blots were measured by ImageJ software (NIH, Bethesda, MD, USA).

### Direct immunofluorescence staining of F‐Actin

To analyse the effect of IL‐6 classic and trans‐signalling on cytoskeleton arrangement, podocytes were cultured on glass plates in a 24‐well plate. After pre‐treated with IL‐6 neutralizing antibody or recombinant sgp130 for 1 hr or transfected with IL‐6R shRNA or scrambled shRNA for 48 hrs, the podocytes were exposed to HG for 24 hrs. The staining and measurement procedures were carried out as we described previously 30.

### Statistical analysis

The data displayed on the graphs are mean values, with error bars representing the standard error of the mean (S.E.M). Significant differences among multiple groups were determined using an anova followed by Student–Newman–Keuls *post ho*c tests. *P* < 0.05 was considered to indicate statistical significance.

## Results

### IL‐6 classic signalling and trans‐signalling are both activated in diabetic kidney

**Figure 1:**
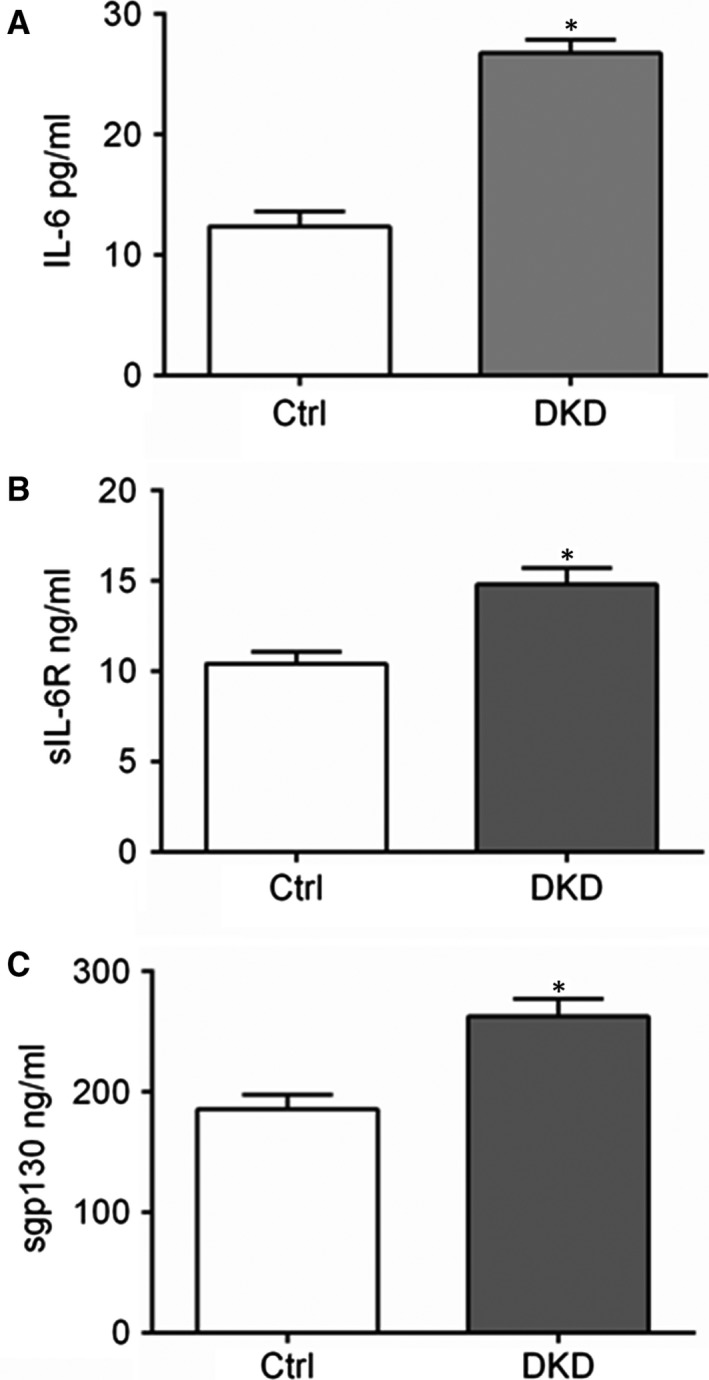
Circulatory IL‐6, sIL‐6R and sgp130 abundance is increased in DKD patients. ELISA analysis data showing IL‐6 (**A**), sIL‐6R (**B**) and sgp130 (**C**) levels in serum samples from healthy controls (Ctrl) and DKD patients (DKD). *n* = 10, *P < 0.05 *versus* Ctrl.

**Figure 2:**
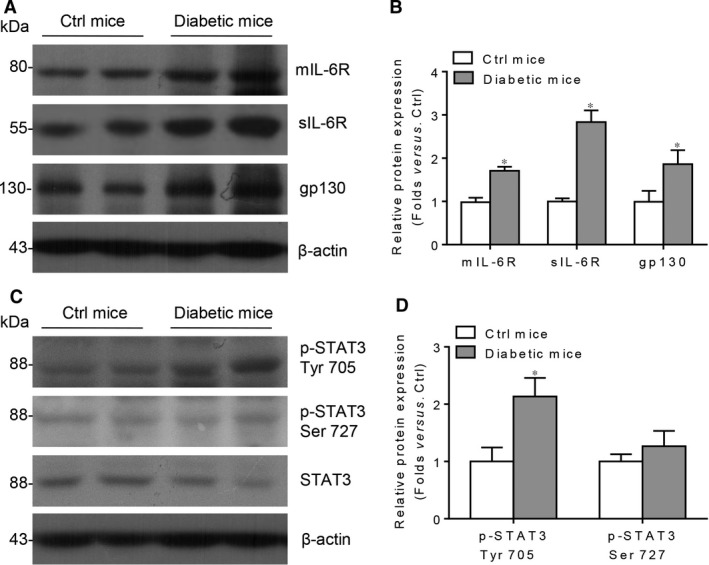
Local classic signalling and trans‐signalling of IL‐6 are activated in kidney from diabetic mice. (**A**) Western blotting analysis and (**B**) summarized data presenting the protein expression of mIL‐6R, sIL‐6R and gp130 in the kidney cortex lysates from control (Ctrl) mice and diabetic mice. (**C**) Western blotting analysis and (**D**) summarized data presenting the protein expression of p‐STAT3 Tyr 705, p‐STAT3 Ser 727 and total STAT3 in the kidney cortex lysates from Ctrl mice and diabetic mice. *n* = 6, *P < 0.05 *versus* Ctrl mice.

To investigate the role of IL‐6 signalling in DKD, we measured the levels of IL‐6, sIL‐6R and sgp130 in the serum from DKD patients and their controls. ELISA analysis showed that the circulatory IL‐6, sIL‐6R and sgp130 levels are significantly higher in the DKD patients compared with the healthy controls (Fig. 1A–C). Consistently, by immunoblotting, we found that in STZ‐induced diabetic mice kidney cortex, the protein levels of mIL‐6R, sIL‐6R and gp130 are significantly increased (Fig. 2A,B). Accordingly, a well‐known IL‐6 downstream signalling STAT3 is activated by phosphorylation on its Tyr 705 residue, other than Ser 727 residue (Fig. 2C,D).

### The expression of mIL‐6R and gp130 is up‐regulated in HG‐cultured podocytes

**Figure 3:**
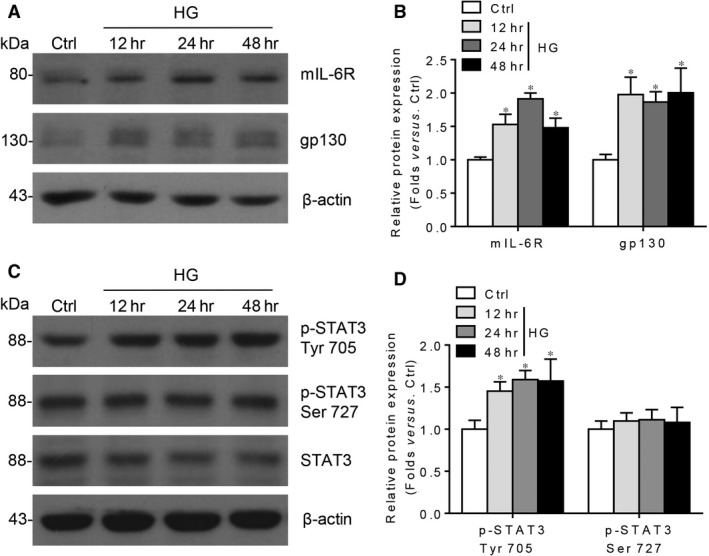
The expression of mIL‐6R and gp130 is up‐regulated in HG‐cultured podocytes. (**A**) Western blotting analysis and (**B**) summarized data presenting the protein expression of mIL‐6R and gp130 in cultured podocytes treated with osmotic control (Ctrl) or high glucose (HG) for indicated times. (**C**) Western blotting analysis and (**D**) summarized data presenting the protein expression of p‐STAT3 Tyr 705, p‐STAT3 Ser 727 and STAT3 in cultured podocytes treated with HG for indicated times. *n* = 5, *P < 0.05 *versus* Ctrl.

As we known, podocytes are the only glomerular residential cells that can transduce both classic and trans‐signalling of IL‐6 and they are also the key component of DKD pathogenesis; next we assess the roles of IL‐6 signalling in HG‐stimulated podocyte. Our data showed that mIL‐6R and gp130 protein levels are increased with HG stimulation in a time‐dependent manner (Fig. 3A,B). Meanwhile, HG treatment increased STAT3 Tyr 705 phosphorylation, without an impact on Ser 727 residue (Fig. 3C,D). Thus, it is proposed that under the HG stimulation, IL‐6‐STAT3 Tyr 705 signalling is activated.

### Simultaneously blocking IL‐6 classic and trans‐signalling attenuates podocyte injury induced by HG

**Figure 4:**
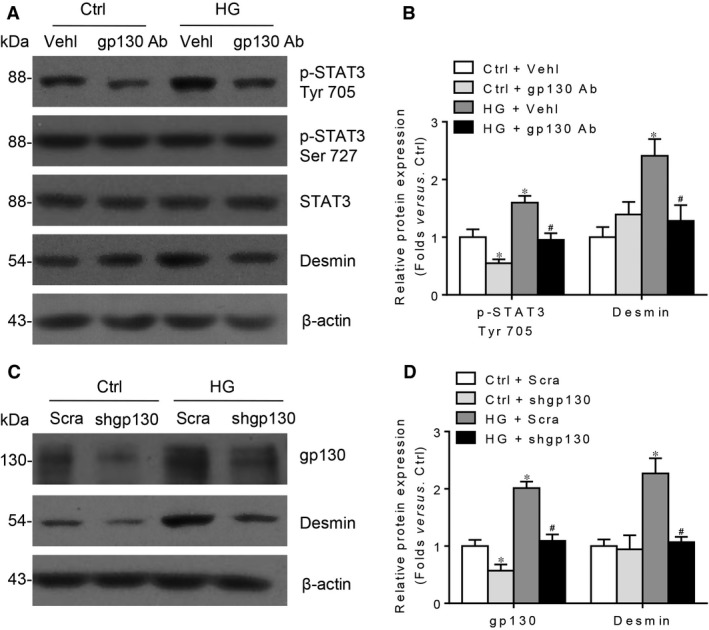
Intervening gp130 to block the entire IL‐6 signalling attenuates HG‐induced podocyte injury. (**A**) Western blotting analysis and (**B**) summarized data presenting the protein expression of p‐STAT3 Tyr 705, p‐STAT3 Ser 727, STAT3 and desmin in HG‐stimulated podocytes pre‐treated without or with gp130 neutralizing antibody (gp130 Ab) for 1 hr (p‐STAT3 Ser 727 summarized data not presented, *P* > 0.05). *n* = 5. Vehl: vehicle. *P < 0.05 *versus* Ctrl + Vehl; ^#^ *P* < 0.05 *versus* HG + Vehl. (**C**) Western blotting analysis and (**D**) summarized data presenting the protein expression of gp130 and desmin in HG‐stimulated podocytes without or with gp130 shRNA transfection. *n* = 6. Scra: scrambled shRNA; shgp130: gp130 shRNA. *P < 0.05 *versus* Ctrl + Scra; ^#^ *P* < 0.05 *versus* HG + Scra.

**Figure 5:**
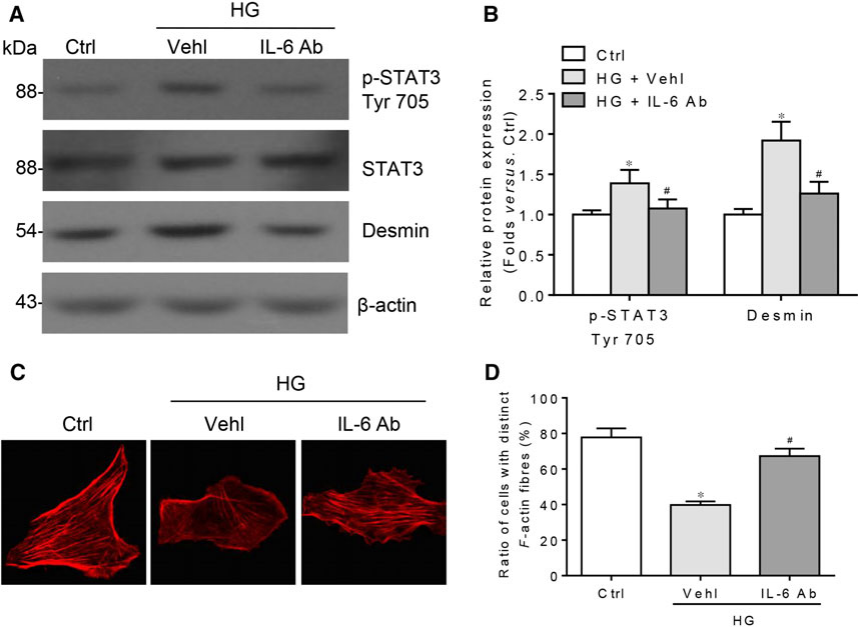
IL‐6 neutralizing antibody attenuates HG‐induced podocyte injury. (**A**) Western blotting analysis and (**B**) summarized data presenting the protein expression of p‐STAT3 Tyr705, STAT3 and desmin in HG‐stimulated podocytes pre‐treated without or with IL‐6 neutralizing antibody (IL‐6 Ab) for 1 hr. *n* = 6. *P < 0.05 *versus* Ctrl; ^#^ *P* < 0.05 *versus* HG + Vehl. (**C**) Microscopic images of F‐actin by rhodamine–phalloidin staining and (**D**) summarized data of the percentage of cells with distinct, longitudinal F‐actin fibres. Scoring was determined from 100 podocytes on each slide. *n* = 3, *P < 0.05 *versus* Ctrl; ^#^ *P* < 0.05 *versus* HG + Vehl.

Gp130 is a critical component for either classic or trans‐signalling of IL‐6, and interrupting gp130 can concurrently affect these two signalling pathways. By utilizing gp130 neutralizing antibody or gp130 shRNA to entirely inhibit IL‐6 signalling, we analysed the roles of the whole IL‐6 signalling during HG‐initiated podocyte damage. Our data indicated that blocking the whole IL‐6 pathway markedly depresses HG‐induced upregulation of desmin, a sensitive and specific indicator for podocyte damage 31. Above response is along with the decreased Tyr 705 phosphorylation of STAT3, not the change of Ser 727 (Fig. 4A–D). Consistently, utilizing IL‐6 neutralizing antibody to completely interrupt IL‐6 signalling can significantly minimize desmin abundance, Tyr 705 phosphorylation of STAT3 as well as the loss and rearrangement of F‐actin in HG‐treated podocyte (Fig. 5A–D). These findings suggest that the whole IL‐6 signalling implicates in HG‐induced podocyte injury, correlating with the Tyr 705 residue phosphorylation of STAT3. Next, we are interested in the individual roles of IL‐6 classic and trans‐signalling in HG‐induced podocyte injury.

### Genetic deletion of IL‐6R alleviates HG‐induced podocyte injury

**Figure 6:**
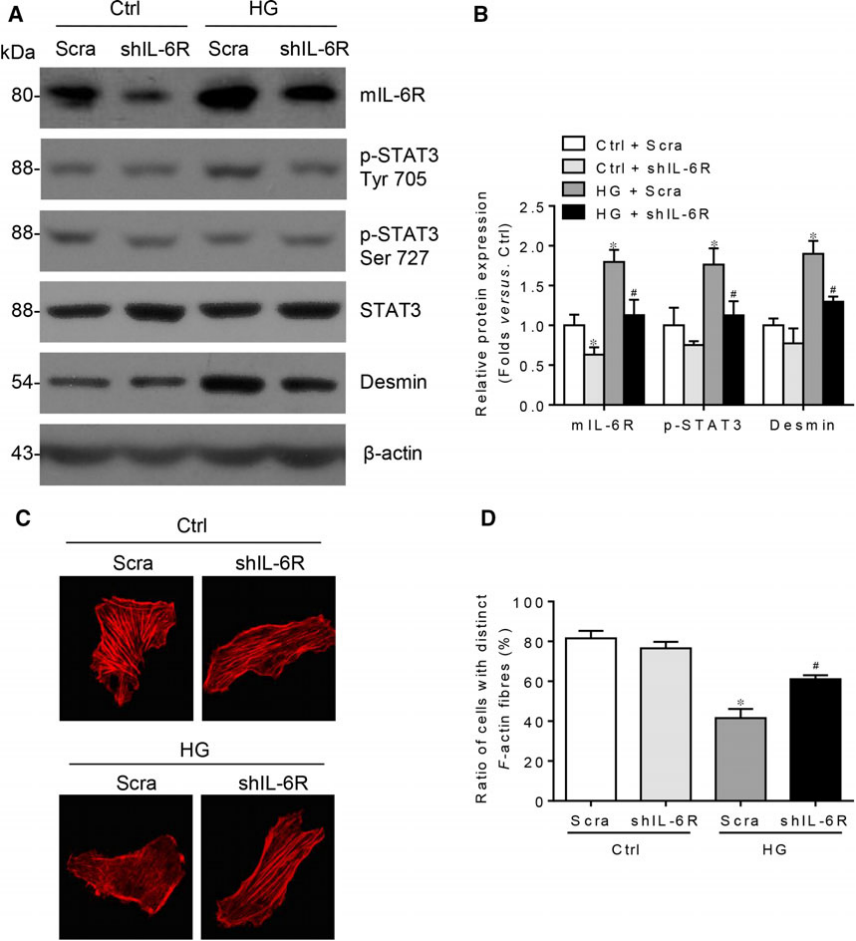
Genetic deletion of IL‐6R alleviates HG‐induced podocyte injury. (**A**) Western blotting analysis and (**B**) summarized data presenting the protein expression of p‐STAT3 Tyr 705, p‐STAT3 Ser 727, STAT3 and desmin in HG‐stimulated podocytes without or with IL‐6R shRNA transfection (p‐STAT3 Ser 727 summarized data not presented, *P* > 0.05). *n* = 6. shIL‐6R: IL‐6R shRNA. *P < 0.05 *versus* Ctrl + Scra; ^#^ *P* < 0.05 *versus* HG + Scra. (**C**) Microscopic images of F‐actin by rhodamine–phalloidin staining and (**D**) summarized data of the percentage of cells with distinct, longitudinal F‐actin fibres. Scoring was determined from 100 podocytes on each slide. *n* = 3, *P < 0.05 *versus* Ctrl + Scra; ^#^ *P* < 0.05 *versus* HG + Scra.

To demonstrate the role of mIL‐6R‐mediated classic signalling in HG‐induced podocyte injury, IL‐6R shRNA was applied. As we shown in Figure 6A–D, IL‐6R shRNA transfection lessens podocyte injury, manifesting as the reduced desmin and preserved F‐actin staining. Meanwhile, the phosphorylation of STAT3 on Tyr 705, but not Ser 727 residue, is dampened by IL‐6R shRNA transfection. Therefore, genetic deletion of IL‐6R can significantly alleviate HG‐induced podocyte injury.

### Inhibition of IL‐6 trans‐signalling ameliorates HG‐mediated podocyte injury

**Figure 7:**
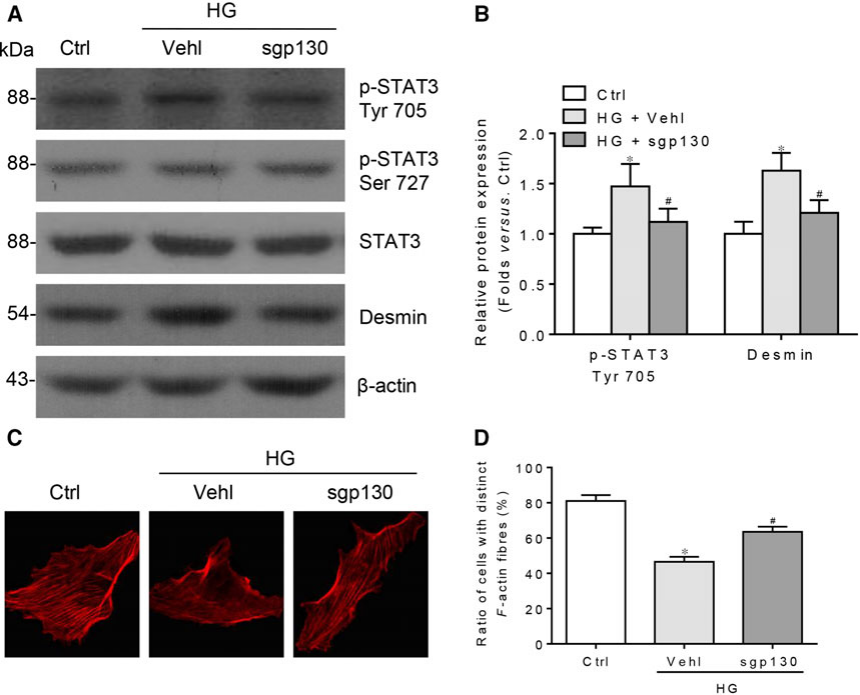
Inhibition of IL‐6 trans‐signalling alleviates HG‐induced podocyte injury. (**A**) Western blotting analysis and (**B**) summarized data presenting the protein expression of p‐STAT3 Tyr 705, p‐STAT3 Ser 727, STAT3 and desmin in HG‐stimulated podocytes pre‐treated without or with recombinant soluble gp130 (sgp130) for 1 hr (p‐STAT3 Ser 727 summarized data not presented, *P* > 0.05). *n* = 5, *P < 0.05 *versus* Ctrl; ^#^ *P* < 0.05 *versus* HG + Vehl. (**C**) Microscopic images of F‐actin by rhodamine–phalloidin staining and (**D**) summarized data of the percentage of cells with distinct, longitudinal F‐actin fibres. Scoring was determined from 100 podocytes on each slide. *n* = 3, *P < 0.05 *versus* Ctrl; ^#^ *P* < 0.05 *versus* HG + Vehl.

And then we investigate the role of IL‐6 trans‐signalling in HG‐induced podocyte injury. Recombinant sgp130 was used to block the IL‐6 trans‐signalling, without affecting IL‐6 classic signalling. As shown in Figure 7A–D, specific intervention of IL‐6 trans‐signalling by sgp130 could obviously alleviate HG‐induced podocyte impairment along with the reduced STAT3 Tyr 705 phosphorylation.

### Either activation of IL‐6 classic or trans‐signalling aggravates podocyte damage

**Figure 8:**
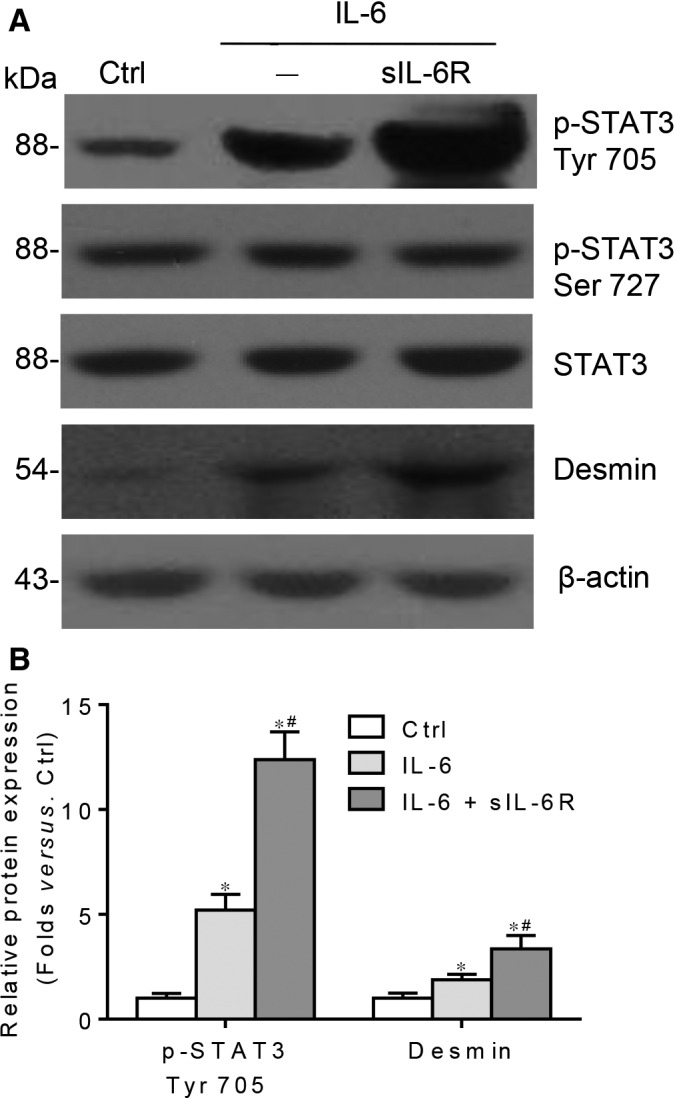
Either activation of IL‐6 classic or trans‐signalling aggravates podocyte damage. (**A**) Western blotting analysis and (**B**) summarized data presenting the protein expression of p‐STAT3 Tyr 705, p‐STAT3 Ser 727, STAT3 and desmin in podocytes treated with IL‐6 or IL‐6‐sIL‐6R (p‐STAT3 Ser 727 summarized data not presented, *P* > 0.05). *n* = 6, *P < 0.05 *versus* Ctrl; ^#^ *P* < 0.05 *versus* IL‐6.

Above findings collectively indicate that IL‐6 classic and trans‐signalling are detrimental for HG‐treated podocyte. Here, IL‐6 is applied to activate the classic signalling of IL‐6, while complex of IL‐6‐soluble IL‐6R is used to activate the trans‐signalling of IL‐6 in vitro stimulation study. As shown in Figure 8A, B, activation of IL‐6 classic signalling in podocytes by IL‐6 significantly increases the phosphorylation of STAT3 on Tyr 705 residue and leads to podocyte damage. Meanwhile, the complex of IL‐6‐sIL‐6R stimulates the STAT3 Tyr 705 phosphorylation and desmin expression to a much higher degree compared with IL‐6. However, there was no change of Ser 727 phosphorylation of STAT3 in either IL‐6 classic or trans‐signalling activation group.

## Discussion

It has been reported that circulatory IL‐6 level is correlated with microvascular complication in type 1 and type 2 diabetic patients 11, 12, 14, and higher serum IL‐6 and sIL‐6R concentrations are observed in type 2 diabetic patients with retinopathy compared with non‐diabetic subjects 12. In current study, we found that the circulatory levels of IL‐6, sIL‐6R and sgp130 are all elevated in DKD patients compared with healthy controls. Moreover, the expression of mIL‐6R, sIL‐6R and gp130 protein is enhanced in kidney cortex of STZ‐induced diabetic mice. These observations indicate that under diabetic status not only in the circulation but also in local kidney, the IL‐6 classic (*via* mIL‐6R) and trans‐signalling (*via* sIL‐6R) are simultaneously activated, and the increased circulatory sgp130 level might represent a compensatory mechanism to limit the activation of IL‐6/sIL‐6R pathway.

On the surface of target cells, IL‐6 initially binds to the mIL‐6R, then the complex of IL‐6/mIL‐6R integrates with the membrane gp130, following the dimerization of gp130 and finally leading to the activation of intracellular signalling. This activation process is termed as classic signalling of IL‐6; however, it is worthwhile to note that only a few cell types, such as macrophages, neutrophils and hepatocytes, express mIL‐6R and therefore can transduce IL‐6 classic pathway. Meanwhile, IL‐6 is able to activate intracellular signalling through soluble IL‐6R, which mainly derived from shedding of the ectodomain of mIL‐6R by disintegrin and metalloproteases (ADAMs) in human. The IL‐6/sIL‐6R complex could bind to the ubiquitously expressed membranous gp130 with the same affinity as IL‐6/mIL‐6R complex, consequently initiates the so‐called trans‐signalling of IL‐6. These two signalling pathways usually exert distinct roles in different diseases. It was reported that IL‐6 classic signalling but not IL‐6 trans‐signalling is responsible for concanavalin A‐mediated liver damage 32. Contradictorily, in idiopathic pulmonary fibrosis, trans‐signalling of IL‐6 is activated and specific disruption of it can significantly attenuate pulmonary fibrosis 33. While in acute kidney disease, IL‐6 trans‐signalling activation is shown to play a beneficial role during lesion resolution 22. Therefore, under different pathologic stimuli, IL‐6 classic and trans‐signalling initiate dissimilar pathogenic events.

It is well described that podocyte is critical for the function of glomerular filtration barrier, and the abnormalities of it are the major event involving in the onset and progression of DKD. More intriguingly, podocyte is the only glomerular resident cell that can transduce both the classic and trans‐signalling of IL‐6. Thus, we are interested in exploring the role of IL‐6 classic and trans‐signalling in podocyte impairment during hyperglycaemia, especially their individual roles. In our experimental designs, the gp130 antibody, gp130 shRNA and IL‐6 antibody are used as the blockers of the entire IL‐6 signalling events, both the classic and trans‐signalling. Meanwhile, IL‐6R shRNA is applied to interrupt the classic signalling of IL‐6 and the sgp130, a natural receptor of IL‐6‐sIL‐6R complex competitively preventing their binding with the membrane‐bound gp130, is applied to block IL‐6 trans‐signalling without affecting the classic signalling 34, 35, 36, 37, 38. Our data show that simultaneously inhibiting both classic and trans‐signalling markedly minimizes HG‐induced podocyte injury, presenting as the reduced desmin levels as well as the attenuated F‐actin loss and rearrangement. Moreover, out of our expectation, individually blocking classic or trans‐signalling can also significantly alleviate podocyte damage. Thus, it suggests that the axis of both IL‐6/IL‐6R and IL‐6/gp130 is harmful to podocyte under hyperglycaemia. To further confirm our results, we stimulated IL‐6 classic or trans‐signalling by IL‐6 or IL‐6‐sIL‐6R complex respectively in vitro, and we found both exhibit detrimental effects on podocytes; however in the latter condition, the damage is more severe which may be related to the relative less quantity of mIL‐6R compared with gp130 on cell membrane. Although by blocking studies, we cannot entirely rule out the possibility that IL‐6R shRNA transfection will reduce sIL‐6R generation and consequently exert an impact on IL‐6 trans‐signalling; however *via* activation experiments, we can still get evidence to support the opinion that IL‐6 classic signalling alone can trigger podocyte damage. In our study, gp130 is injurious in DKD; unlikely, leukaemia inhibitory factor (LIF), another gp130 family cytokine, has been reported to attenuate high glucose‐induced podocyte apoptosis, suggesting a protective role of gp130 family member in podocyte injury during DKD 39. In addition, other gp130‐type cytokines, including IL‐27 and IL‐31, are associated with the pathogenesis of diabetic retinopathy 40, 41. Thus, the function of gp130 families is heterogeneous.

STAT3 is the major downstream signalling of IL‐6 and plays an essential role in hyperglycaemia‐initiated glomerular and podocyte injury. Lu *et al*. genetically generated mice models that contain 75% and 25% STAT3 activity, respectively; the latter with less STAT3 activity are characterized by significant minimized proteinuria, mesangial expansion, glomerular cell proliferation and macrophage infiltration compared with former ones under the diabetic status 42. Additionally, in mice study of nephrotoxic serum‐induced glomerulonephritis, specific podocyte STAT3 deletion mitigates crescent formation and preserves renal function than their control ones 43. STAT3 contains several different phosphorylation sites, and it is generally believed that Tyr 705 phosphorylation is essential for STAT3 transcriptional activity. For example, the phosphorylation of Tyr705 is indispensable for STAT3‐mediated embryonic stem cell self‐renewal, while Ser 727 phosphorylation is not required 25. On the other hand, the role of Ser 727 phosphorylation is controversial, and it may have a positive or negative impact on the activity of STAT3 25, 44, 45, 46. As for in prostate tumorigenesis, Ser 727 phosphorylation is sufficient to activate STAT3, whereas it is not true in osteoclast precursors 26. So at beginning, we speculated that there might be a possible association between the phosphorylation site of STAT3 and the activation modes of IL‐6 signalling, which subsequently plays different roles in podocyte impairment during hyperglycaemia. Nevertheless, in diabetic mice and HG‐treated podocytes, only enhanced Tyr 705 phosphorylation is detected without alteration of Ser 727 phosphorylation. Moreover, regulating the classic and trans‐signalling of IL‐6 also has no impact on Ser 727 phosphorylation, while it does affect the Tyr 705 phosphorylation accordingly.

Taken together, our study demonstrated that both the IL‐6 classic signalling and trans‐signalling are activated and play a detrimental role in podocyte injury during hyperglycaemia *via* STAT3 Tyr 705 phosphorylation. Moreover, previous study shows that in glomerular mesangial cells, IL‐6 trans‐signalling promotes monocyte chemoattractant protein 1 (MCP‐1), IL‐8 and macrophage inflammatory protein‐1 alpha (MIP‐1 alpha) production, which consequently results in mesangial proliferation and inflammatory response 21. IL‐6 deficiency mouse is protected from angiotensin II‐initiated endothelial dysfunction and hypertrophy 47. These data suggest that IL‐6 signalling not only plays a detrimental role in podocyte dysfunction, but also attributes to the injury of other two glomerular resident cells. Therefore, blocking IL‐6 signalling seems to be an ideal strategy to cure DKD. Tocilizumab, a IL‐6 receptor inhibitor, has been approved for treatment in patients with certain autoimmune disorders, such as rheumatoid arthritis, juvenile idiopathic arthritis and Castleman's disease 48. More excitingly, tocilizumab is reported to successfully improve renal function and attenuate proteinuria in rheumatoid arthritis and Castleman's disease patient with renal failure 49, 50. Taken together, intervening IL‐6 signalling should be a more promising preventive and therapeutic target for DKD in the near future.

## Conflict of interest

The authors declare that there is no conflicts of interest regarding the publication of this paper.
